# *Trypanosoma rangeli* infection impairs reproductive success of *Rhodnius prolixus*

**DOI:** 10.1017/S0031182022001470

**Published:** 2023-01

**Authors:** Bruna Duarte da Silva, Alessandra Aparecida Guarneri

**Affiliations:** Vector Behavior and Pathogen Interaction Group, Instituto René Rachou, Fundação Oswaldo Cruz-FIOCRUZ, Belo Horizonte, Minas Gerais, Brazil

**Keywords:** Fecundity, fertility, longevity, parasite–vector interaction, *Rhodnius prolixus*, *Trypanosoma rangeli*

## Abstract

*Trypanosoma rangeli* is a protozoan that infects triatomines and mammals in Central and South America. Although it does not cause disease to humans, this parasite produces different levels of pathogenicity to its invertebrate host, mainly in species of the genus *Rhodnius*. In this study, we followed *T. rangeli*-infected and uninfected pairs throughout their adult lives and measured the amount of blood ingested, number of eggs laid, number of eggs hatched and proportion of infertile eggs, as well as female life expectancy. We found that all reproductive parameters were drastically decreased during infection, mainly due to the reduced amount of blood the infected insects ingested throughout their lives. Reproductive parameters were also affected by the reduction of the life expectancy of infected females, as survival was positively correlated with the number of eggs laid. The strategies used by the parasite to be transmitted are discussed in view of the pathological effects it causes in the insect.

## Graphical abstract

**Figure:**
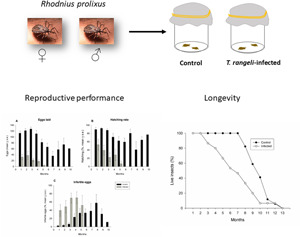

## Introduction

*Trypanosoma rangeli* is a digenetic parasite that infects triatomines and mammals in Central and South America (Guhl and Vallejo, 2003). The parasite does not cause disease in mammals, including humans, and its development in vertebrate hosts is still unknown. *In vitro* studies suggested that *T. rangeli* multiplication can occur in monocytes, but they failed to show replicative forms (Osorio *et al*., 1995; Eger-Mangrich *et al*., 2001). A more recent study suggested that it can multiply in secondary lymphoid organs, as DNA and live parasites were found in mice's spleen and lymph nodes 30 days post-infection (Ferreira *et al*., 2020). *Trypanosoma rangeli* infects triatomine species of different genera, but only completes its development, producing infective forms, in *Rhodnius* spp. Nevertheless, even when infecting *Rhodnius*, salivary gland colonization depends on the genetic background of the parasite and the host, which suggests a co-evolutionary association between *T. rangeli* isolates and their sympatric vectors (Maia da Silva *et al*., 2007; Urrea *et al*., 2011).

The development of *T. rangeli* in susceptible *Rhodnius* species begins when the triatomine feeds on an infected mammal and ingests the parasite. Blood trypomastigote forms start to differentiate into epimastigotes, the multiplicative forms, a few hours after reaching the anterior midgut (Ferreira *et al*., 2018). Epimastigotes colonize the entire intestinal tract of the insect within 1–2 weeks after the infection started (Ferreira *et al*., 2018). The infection is restricted to the intestine for a significant proportion of infected bugs. The mechanisms by which the parasite reaches the haemolymph are unknown, but the perimicrovillar membranes seem to function as a mechanical blockage (Gomes *et al*., 1999). The parasites reaching the haemocoel multiply freely in the haemolymph and then invade the salivary glands where metacyclogenesis occurs. Parasites will be transmitted when the insect feeds on a mammal, being inoculated into the host together with the saliva.

*Trypanosoma rangeli* can produce massive infections in the insect, which will generate different levels of pathogenic effects. Increased mortality rates, delay in development and post-ecdysis deformations are all reported effects in *T. rangeli*-infected *Rhodnius* (reviewed in Guarneri and Lorenzo, 2017; Guarneri and Schaub, 2021). We have previously shown that *T. rangeli* decreases the fecundity of newly moulted adults of *Rhodnius prolixus* by interfering with the amount of blood females use in egg production (Fellet *et al*., 2014). In the present study, we followed a group of infected and uninfected pairs of *R. prolixus* during all their adult lives and evaluated the effects of *T. rangeli* infection on different parameters such as fecundity, fertility and life expectancy.

## Material and methods

### Triatomines

*Rhodnius prolixus* used in this study were obtained from a long-established colony in our laboratory (originally collected in Honduras in the 1990s). Insects were fed citrated rabbit blood on a monthly basis obtained from CECAL (Fiocruz, Rio de Janeiro, Brazil) offered through an artificial feeder at 37°C, alternating with blood from anaesthetized chickens [intraperitoneal injections of a mixture of ketamine (20 mg kg^−1^; Cristália, Brazil) and detomidine (0.3 mg kg^−1^; Syntec, Brazil)] and mice [intraperitoneal injections of a mixture of ketamine (150 mg kg^−1^; Cristália) and xylazine (10 mg kg^−1^; Bayer, Brazil)]. The colony was maintained at 27 ± 1°C, 51 ± 7% RH, and exposed to a natural illumination cycle. All experiments using live animals were performed following FIOCRUZ guidelines on animal experimentation and were approved by the Ethics Committee on Animal Use (CEUA/FIOCRUZ) under the approved protocol number LW 8/17.

### Parasites

The CHOACHI strain, originally isolated from naturally infected *R. prolixus* salivary glands (Schottelius, 1987), was used to infect insects. The parasites were cultured by twice a week passages in liver-infusion tryptose medium supplemented with 15% fetal bovine serum, 100 mg mL^−1^ streptomycin and 100 units mL^−1^ penicillin. The maintenance of parasites exclusively in culture medium generated parasites unable to colonize the salivary glands (Rodrigues *et al*., 2016). We used this population to guarantee a sufficient number of infected adults since, in our model, nymphs infected with parasites able to colonize the salivary glands hardly reach the adult phase (data not shown).

### Insect infection

*Trypanosoma rangeli* infection was performed as described by Guarneri (2020). Heat-inactivated (56°C, 30 min) citrated rabbit blood containing culture epimastigotes (1 × 10^6^ parasites mL^−1^) was offered in an artificial feeder for third instar nymphs. Seven days after moulting to the fourth instar, 1 *μ*L of sterile phosphate-buffered saline (PBS) (0.15 M sodium chloride in 0.01 M sodium phosphate, pH 7.4) containing 2.5 × 10^4^ epimastigotes mL^−1^ was inoculated into the coelomic cavity of each nymph. A 50 *μ*L syringe (Hamilton Company, USA, needle 13 × 3.3; ½”) connected to a dispenser (model 705, Hamilton Company) was used to inoculate the parasites. This inoculum is necessary to ensure systemic infections in all individuals with intestinal infections (reviewed in Guarneri and Lorenzo, 2017; Guarneri and Schaub, 2021). One day after inoculation, insects were fed on anaesthetized mice. A sample of haemolymph collected from one of the tarsi was examined to confirm haemocoelomic infection. Insects in the control group were fed heat-inactivated citrated rabbit blood, inoculated with sterile PBS, and a haemolymph sample was collected at the same period as the infected group. After the infection procedure, anaesthetized mice were used as hosts for all other feedings. The experimental groups were kept in a chamber maintained at 26 ± 0.5°C and 12:12L/D. After moulting to the fifth instar, nymphs were sexed, and males and females were maintained in separated containers to prevent copula in newly moulted adults.

Two weeks after the imaginal moult, the adults were fed anaesthetized mice, and 1 week later, the insects were arranged in pairs (*n* = 18 and 15, for infected and uninfected groups) that were maintained in separate plastic vials (5.5 × 8.0 cm). Each vial contained a circular piece of filter paper and a cardboard strip as substrate and was closed with a cloth. Weekly, anaesthetized mice were offered to the insects for 1 h. Insects were individually weighed before and immediately after removing the mouse. The eggs produced by each pair were collected weekly and transferred to plastic plates. The following parameters were recorded: (a) amount of blood ingested; (b) the number of eggs laid; (c) egg hatching rate; (d) the percentage of infertile eggs (the absence of the embryo was confirmed by egg examination under a stereoscopic microscope); (e) life expectancy of females. The pairs were followed until the death of the female.

### Statistical analyses

Multivariate generalized estimating equation (GEE) models were used to analyse reproductive parameters as the data included repeated measures and did not fit a parametric distribution. GEE models with the identity link function were adjusted considering the amount of blood ingested, the number of eggs laid, hatching rates and the percentage of infertile eggs as continuous outcomes. Infection status and time were used as covariates in all models. Sex was used as a third covariate when the model was adjusted to the amount of blood ingested. An exchangeable working correlation structure was used in the analyses. Parameter estimates were presented as coefficients (Coeff) with 95% confidence intervals (CI). Survival curves were compared through log-rank (Mantel–Cox) test. Spearman's correlation was used to test the correlation between life expectancy and eggs laid. Data analyses were conducted using the statistical software JASP (Version 0.16.1) and R version 4.1.2 (R Core Team 2021, R Foundation of Statistical Computing, Vienna, Austria). GEE analysis was conducted using the package ‘geepack’ (Halekoh *et al*., 2006). All analyses considered the differences statistically significant when *P* < 0.05.

## Results

**Fig. 1.**
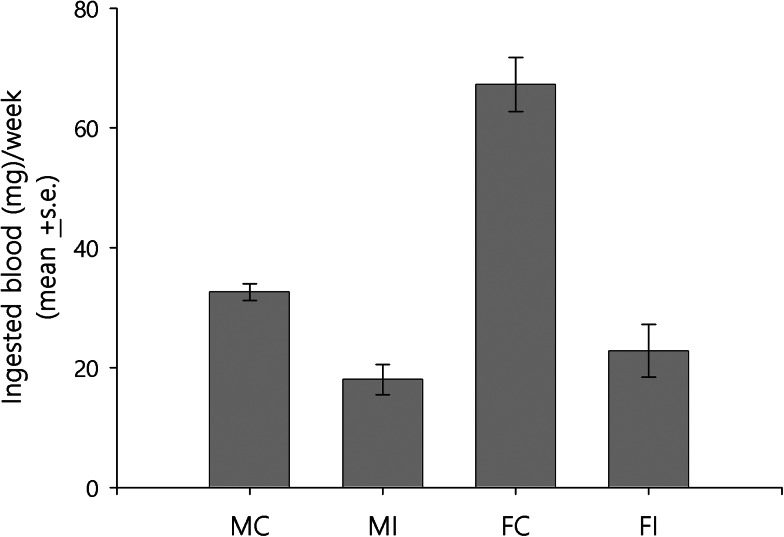
Amount of blood ingested *by Rhodnius prolixus* adults is affected by sex and *Trypanosoma rangeli* infection. Bars show the mean ± s.e. of the weekly amount of blood ingested (see Table 1 for significance values; *n* = 8 males and *n* = 8 females for control group, *n* = 13 males and *n* = 13 females for infected group). M, male; F, female; C, control; I, infected.

**Fig. 2.**
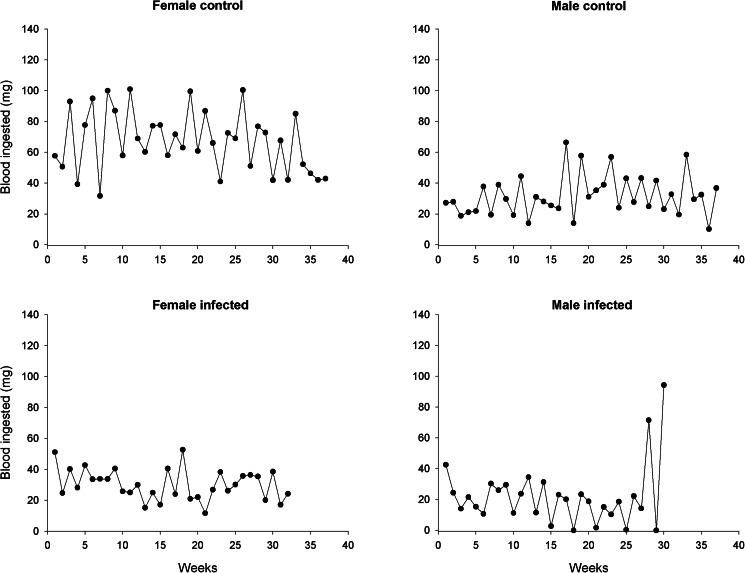
Blood ingested throughout adult life by *Rhodnius prolixus* infected or not with *Trypanosoma rangeli*. Data are shown as the weekly average amount of blood ingested. Female control, *n* = 9; male control, *n* = 9; female infected, *n* = 14; male infected, *n* = 12.

The weight of newly moulted adults was similar for infected and uninfected individuals for both sexes (*t*-test, d.f. = 14, *P* > 0.05 for both sexes; *n* = 8 males and *n* = 8 females for control group, *n* = 13 males and *n* = 13 females for infected group). The amount of blood ingested by the adults was affected by infection and sex (Fig. 1; Table 1). Infected adults ingested less blood than uninfected ones [Table 1; Coeff (95% CI) = −33.580 (−45.578 to −21.581); *P* < 0.001]. Regarding sex, males ingested less blood than females [Table 1; Coeff (95% CI) = −44.763 (−55.006 to −34.519); *P* < 0.001]. The adjusted model showed a significant interaction of time with infection [Table 1; Coeff (95% CI) = −0.500 (−0.952 to −0.047); *P* = 0.03] and sex [Table 1; Coeff (95% CI) = 0.536 (0.128–0.944); *P* = 0.01]. Significant differences over time between infected and uninfected individuals were observed only for females. Setting time at 4 weeks, the mean amount of blood ingested by infected females was 35.6 mg less than that of uninfected ones [95% CI (−52.1 to −33.2)]. The differences became larger as time increased. For example, at 7 weeks, the difference between the 2 groups was 37.1 mg [95% CI (−47.5 to −26.7)]. Regarding sex, differences over time were only observed in the control group. At 4 weeks, the mean amount of blood ingested by males was 42.6 mg [95% CI (−52.1 to −33.2)] less than that of females. At 7 weeks, the difference decreased to 41 mg. Furthermore, as shown in Fig. 2, all groups, except the infected females, showed a tendency of ingesting blood every 2 weeks.

**Table 1.** Variables of the generalized estimating equations GEEs with significant effects on the amount of blood ingested by *Rhodnius prolixus* infected or not with *Trypanosoma rangeli* (*n* = 8 males and *n* = 8 females, for control group, *n* = 13 males and *n* = 13 females for infected group).

Covariates	Multivariate analysis (95% CI)	*P*
Time	−0.034 (−0.344 to 0.275)	0.828
Infection		
Control	*Reference*	
Infected	−33.580 (−45.578 to −21.581)	<0.001
Sex		
Female	*Reference*	
Male	−44.763 (−55.006 to −34.519)	<0.001
Interaction time:infection	−0.500 (−0.952 to −0.047)	0.031
Interaction time:sex	0.536 (0.128–0.944)	0.01
Interaction infection:sex	29.272 (18.083–40.461)	<0.001

**Fig. 3.**
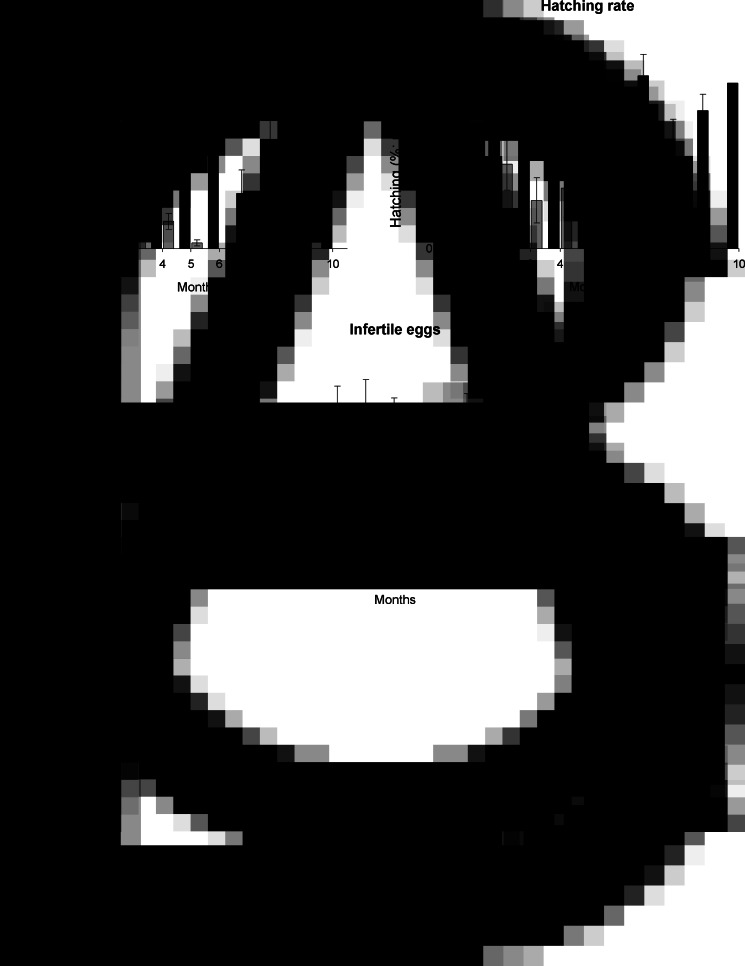
Reproductive success of *Rhodnius prolixus* is highly affected by *Trypanosoma rangeli* infection. (A) Number of eggs. (B) Hatching rates. (C) Infertile eggs (see Table 2 for significance values; *n* = 8 for control group, *n* = 13 for infected group).

All parameters of reproductive development were affected by infection, time and amount of blood ingested (Fig. 3). As expected, the amount of blood ingested positively affected the number of eggs laid (Table 2) [Coeff (95% CI) = 0.002 (0.00–0.004); *P* < 0.027; *n* = 8 for control group, *n* = 13 for infected group]. The coefficient of the comparison between infected and uninfected groups was negative, indicating that infected females produced fewer eggs than the control ones (Table 2). Indeed, uninfected females laid 652.3 ± 53.0 eggs during their lifetime, while infected ones laid only 92.2 ± 28.1. In addition, the model showed a significant interaction between time and infection. Uninfected females remained ovipositing for approximately 7 months (except for 1 pair, where the female laid eggs for 10 months). In the infected group, females stopped laying eggs after 5 months. The coefficient of the interaction between these parameters showed that the differences in the number of eggs laid by infected and uninfected females increased over time (Table 2; Fig. 3A). Eggs laid by infected females showed lower hatching rates [Fig. 3B, Coeff (95% CI) = −30.176 (−57.302 to −3.049; *P* = 0.029)]. Hatching rates also decreased with time [Coeff (95% CI) = −6.859 (−11.727 to −1.991); *P* = 0.006] and were positively affected by the amount of blood ingested [Coeff (95% CI) = 0.140 (0.035–0.244); *P* = 0.009]. The number of infertile eggs increased over time [Fig. 3C; Coeff (95% CI) = 7.702 (2.444–12.959); *P* = 0.004] and was higher in the infected group [Coeff (95% CI) = 30.236 (7.943–52.528); *P* = 0.008]. The amount of blood ingested negatively affected the number of infertile eggs [Coeff (95% CI) = −0.115 (−0.196 to −0.034); *P* = 0.005].

**Table 2.** Variables of the generalized estimating equations GEEs with significant effects on the number of eggs laid by *Rhodnius prolixus* females infected or not with *Trypanosoma rangeli* (*n* = 8 for control group, *n* = 13 for infected group).

Covariates	Multivariate analysis (95% CI)	*P*
Time	−0.058 (−0.131 to 0.016)	0.124
Infection		
Control	*Reference*	
Infected	−0.287 (−0.878 to 0.304)	0.341
Amount of blood ingested	0.002 (0.00–0.004)	0.027
Interaction time:infection	−0.377 (−0.488 to −0.265)	<0.001

**Fig. 4.**
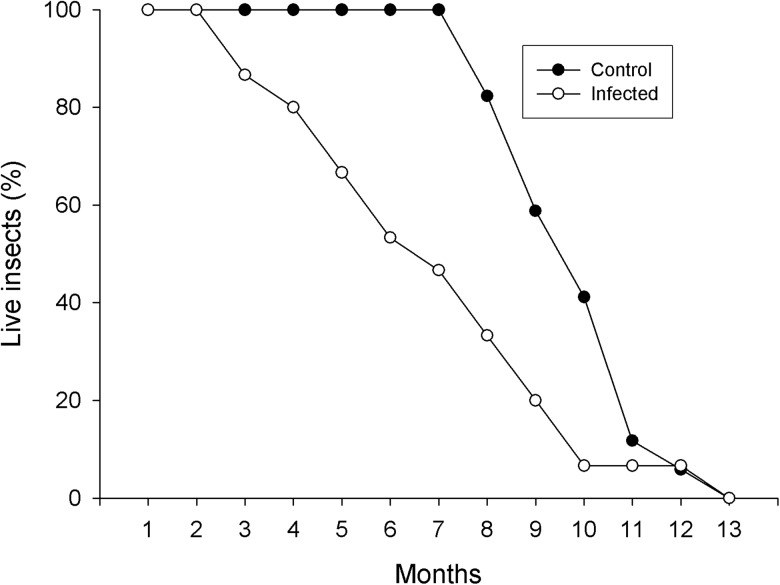
*Trypanosoma rangeli* infection decreases the life expectancy of *Rhodnius prolixus* females (log-rank, *P* = 0.01; *n* = 13 for control group, *n* = 15 for infected group).

**Fig. 5.**
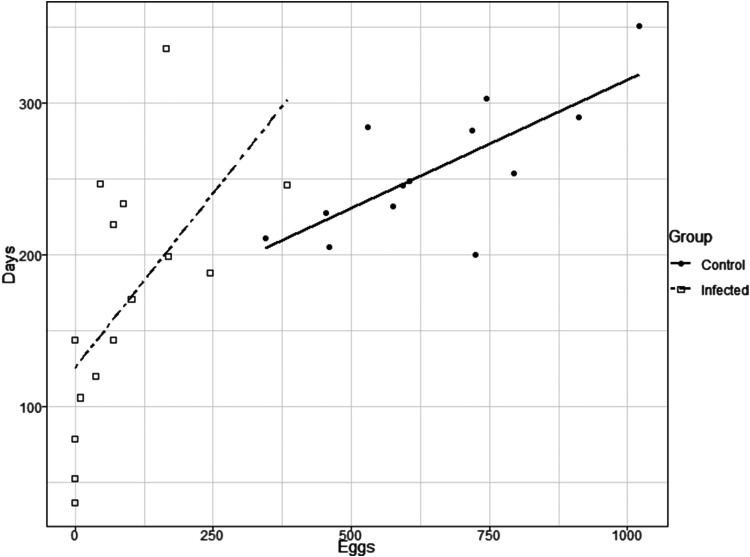
Life expectancy-fecundity trade-off in *Rhodnius prolixus* infected or not with *Trypanosoma rangeli*. The raw plot of the number of eggs that *R. prolixus* females laid against their survival. Filled circles show uninfected female; empty squares represent infected females. The linear regression of triatomine survival against the number of eggs each female laid was plotted using a full line for uninfected females and a dashed line for infected ones (Spearman's *r* = 0.65, *P* = 0.01 for uninfected females; *r* = 0.73, *P* = 0.002 for infected females; *n* = 13 for control group, *n* = 15 for infected group).

The life expectancy of infected females was also affected by *T. rangeli* infection (Fig. 4; log-rank, *P* = 0.01; *n* = 13 for control group, *n* = 15 for infected group). While uninfected females started to die after 7 months, only 33.3% of infected females were still alive in that period. The survival of uninfected and infected females was positively correlated with the number of eggs laid (Fig. 5; Spearman's *r* = 0.65, *P* = 0.01 for uninfected females; *r* = 0.73, *P* = 0.002 for infected females).

## Discussion

Infection by *T. rangeli* severely affected all reproductive parameters evaluated, in addition to decreasing life expectancy rates of *R. prolixus*. Many parasites and pathogens reduce fecundity and host survival (Williamson and Von Wechmar, 1995; Maciel-de-Freitas *et al*., 2011); while some only interfere with fecundity and fertility (Styer *et al*., 2007), others do not promote alterations in any of these parameters (Costanzo *et al*., 2014).

In our model, using a natural vector–parasite combination, we found a ~85% reduction in the number of eggs laid by infected females throughout their adult lives. Furthermore, more than half of these eggs did not hatch, mainly because no embryo developed in them. Blood digestion in *Rhodnius* takes place for about 14 days. After each blood meal, the fat body accumulates triacylglycerol, used for ovary growth and the oogenesis cycle in adult females (Gondim *et al*., 2018). Confirming this, uninfected females presented a tendency of feeding every 2 weeks, which was not observed in infected ones that ingested small quantities of blood on every occasion a mouse was offered. The reduced capacity to ingest blood by infected adults was possibly the most important determinant of fecundity reduction. When *T. rangeli* colonizes *R. prolixus* salivary glands, a 60% reduction in stored proteins is observed, which, in turn, affects the feeding behaviour of infected individuals, making blood feeding less efficient (Grewal, 1956; Añez and East, 1984; Garcia *et al*., 1994; Paim *et al*., 2013). However, it is worth mentioning that we used parasites unable to invade salivary glands, as in our model, insects infected with invasive parasites rarely reach adulthood. Therefore, it will be interesting to evaluate in future studies what other effects of the infection affect the capacity of infected bugs to ingest blood. We previously showed that *T. rangeli* infection reduced the amounts of ingested blood turned into eggs in infected females (Fellet *et al*., 2014). As *T. rangeli* incorporates lipids from the insect (Folly *et al*., 2003), part of the already scarce nutritional resources of females is used by the parasite for its own development, which further reduces the reserves available for egg production. Parasites transmitted vertically have their reproductive success dependent on that of their hosts; therefore, such parasites frequently develop low virulence. Horizontal transmission, in contrast, is less constrained by host condition and the increase in the parasite fitness can evolve at additional costs to the host (Agnew and Koella, 1997; Stewart *et al*., 2005). In this sense, as a parasite that is transmitted only horizontally, reducing adult fecundity would not negatively affect the fitness of *T. rangeli*.

It is well known that there is a trade-off between reproduction and survival, as reproduction imposes costs on females that translate into a decrease in survival rates (Williams, 1966; Tatar, 2010). Therefore, a negative correlation between the number of eggs laid and the life expectancy would be expected, as observed in *Culex pipiens* (Vézilier *et al*., 2012). Interestingly, the infection of *C. pipiens* with *Plasmodium relictum* reduces egg production, which in turn prolongs female survival, reinforcing the idea of reproductive costs (Vézilier *et al*., 2012). In our study, we found that the number of eggs laid by each individual female was positively correlated to its subsequent survival, regardless of the presence of the parasite. This suggests that in *R. prolixus* a reduction in fecundity, such as that promoted by *T. rangeli* infection, does not translate into higher life expectancy. Some species can use the strategy of fecundity compensation when parasite pressure is high by investing more in current reproduction to minimize the fitness loss due to parasitism (Schmid-Hempel, 2021). Therefore, the steeper slope of the fecundity–survival relationship of infected females suggests that certain adjustments in the pattern of oviposition of females may occur during the infection, so these females can guarantee that some viable offspring be produced before they die.

The pathological effects promoted by *T. rangeli* have been widely related. Prolongation of the intermoult period or even interruption of the moulting process, defective ecdysis and increased mortality rates that occur mainly during ecdysis have been observed in different species of *Rhodnius* experimentally infected (Grewal, 1957; Tobie, 1965; Watkins, 1971; Añez, 1984; Cuba Cuba, 1998; Peterson and Graham, 2016; Rodrigues *et al*., 2016). The parasite is also responsible for reducing the microbiota diversity in the anterior midgut of *R. prolixus* (Eberhard *et al*., 2022), which probably affects the insect's fitness. The high parasitaemia in the insect is probably maintained through modulation of the immune system, which is downregulated from the moment the parasite reaches the intestinal tract of the insect (Mello *et al*., 1999; Whitten *et al*., 2001; Gomes *et al*., 2003; Garcia *et al*., 2004; Rolandelli *et al*., 2021).

Given this scenario, the question that arises is, how is such a pathogenic infection maintained in the insect? Considering all the alterations promoted by the parasite, it would be challenging for infected individuals to reach adulthood, and those who did would produce a very small number of offspring. We hypothesize that the drastic effects promoted by the systemic infection are balanced by a decrease in the number of individuals harbouring parasites in the haemolymph and salivary glands, associated with a high transmission efficiency. Despite the low or even unapparent parasitaemia that *T. rangeli* promotes in vertebrate hosts, the infection rates of insects fed on infected mice are around 80% (Ferreira *et al*., 2015). However, the percentage of insects with an intestinal infection that develop a systemic infection normally does not exceed 50% (reviewed in Guarneri and Schaub, 2021). Interestingly, in insects collected in the wild, systemic infection rates are lower, ranging from 1 to 15% (Pifano and Mayer, 1949; D'Alessandro and Mandel, 1969). This low rate of systemic infections would be relevant in the interaction, as intestinal infections are usually less pathogenic (Ferreira *et al*., 2010). The transmission of pathogens by means of biting is highly efficient since pathogens are inoculated into the skin of the host. In the case of *T. rangeli*, an infected *R. prolixus* nymph can release up to 50 000 parasites during a blood meal, causing mice infection rates of 90% (Ferreira *et al*., 2015). In addition, behavioural alterations promoted by the infection make the infected individuals more active, increasing the number of insects that leave the protection of shelters and try to feed on a vertebrate host (Marliére *et al*., 2015, 2022). With elevated transmission rates increased by behavioural changes, a small number of individuals developing systemic infections would be sufficient to maintain the circulation of the parasite. Furthermore, it has been shown that *T. rangeli*-infected insects can transmit parasites to co-specifics when feeding simultaneously on the same host, even if the host is not susceptible to the parasite, such as birds for example (Tobie, 1961; Ferreira *et al*., 2015). This behaviour would increase the number of individuals harbouring parasites in the intestinal tract and the chances of some of them developing systemic infections, ensuring the parasite transmission.

## Data Availability

Data used in this publication can be accessed in the supplementary material.

## Supplementary material

For supplementary material accompanying this paper visit https://doi.org/10.1017/S0031182022001470. click here to view supplementary material
